# Impact of Multi-Disciplinary Team in Implementing Clinical Audit Recommendations to Improve Health Educators’ Practice at Primary Care in Qatar

**DOI:** 10.1177/21501319241279656

**Published:** 2025-03-31

**Authors:** Ansif Pallath Majeed, Kiran Harikumar, Hanan Al Mujalli, Abdul Ali Shah, Noora Alkubaisi, Sharifullah Khan, Faiza Aiman

**Affiliations:** 1 Primary Health Care Corporation, Doha, Qatar

**Keywords:** health educator, SWOT analysis, multi-disciplinary team approach, primary care, clinical audit, quality improvement

## Abstract

**Background:**

Health educators play a vital role in primary care by educating patients on the significance of maintaining good health and preventive measures, especially in the case of chronic illnesses. Evaluating the impact of their efforts is essential to ensure the delivery of high-quality patient care.

**Method:**

A multi-layered process involves pre-intervention analysis of 180 health records, followed by a SWOT analysis to identify necessary interventions, facilitated by a multidisciplinary team to enhance the quality of practice. Subsequently, a post-intervention re-audit is conducted with a sample size of 236 health records to assess any changes in measured parameters.

**Results:**

The initial health education and learning needs assessment compliance was 41% during the baseline evaluation but increased to 81% (*P* = .001) post-intervention. Social history review improved from 54% to 75% (*P* = .198) following the intervention. Mental health screening for anxiety and depression was done on 67% of patients initially, but compliance rose to 98% (*P* ≤ .001) post-intervention. Problem list documentation reached 100%(*P* ≤ .001) compliance post-intervention, up from 44% during the baseline assessment. In the initial evaluation, it was found that 56% of patients were provided with health education and follow-up guidance. After the intervention, this number increased to 79%(*P* = .013). During the baseline assessment, 35% of patients underwent follow-up assessments, including a review of previous goals and their achievement status. This percentage significantly improved to 92%(*P* = .001) after the intervention. Additionally, 47% of patients received a follow-up plan with new goals and instructions before the intervention, but this improved to 90% (*P* = .004) afterwards. The overall compliance rate during the baseline assessment was 51%, which increased to 88% following the intervention, indicating a significant improvement in the health educator’s practice.

**Conclusion:**

The involvement of a multidisciplinary team in implementing multifaceted post baseline recommendations was instrumental in enhancing the overall performance of health educators. The study results underscore the positive impact of this cooperation among different departments on the efficacy of health educators’ practice. The SWOT analysis helped to examine various possibilities for implementing interventions to address existing gaps and enhance the quality of practice through positive changes.

## Introduction

The importance of quality health education cannot be overstated in the realm of contemporary health promotion.^1^ It is instrumental in the treatment of chronic diseases, particularly in older patients. This education not only aids in patients’ comprehension of their conditions but also cultivates a positive approach towards medical advice. Moreover, it alleviates patients’ anxiety and depression, ultimately enhancing their overall quality of life.^2^ Additionally, health education empowers patients to actively participate in their healthcare journey, increasing the likelihood of effective self-management.^3^

A study conducted in 2017 to learn the impact of health education program on the lifestyle habits of middle-aged women at risk of osteoporosis showed that Women in the intervention group who received the health education program had improved physical activity levels, increased daily calcium intake, and increased general knowledge about osteoporosis.^4^ These lifestyle changes may have delayed or even prevented the onset of osteoporosis in some women. Involving vulnerable populations in health education programs that address diabetes, obesity, and even cancer can have significant short- and long-term benefits.^4^

Health educators in primary care settings are vital in informing patients about the significance of maintaining good health and practicing preventive care.^5^ Primary care is considered the initial point of contact for individuals seeking healthcare services and minimize disparities in health among significant population subsets.^6^ Research conducted in primary health centers in Saudi Arabia discovered that the provision of patient education on self-care by certified health educators at primary healthcare facilities can effectively reduce blood sugar levels, decrease hypertension, waist circumference, and mean body mass index enhancing the overall quality of life related to health, in comparison to patients who did not participate in the educational session.^7,8^

Proper documentation plays a vital role in the field of health education.^9^ Maintaining documentation is a customary method of preserving continuous patient care details. It encompasses pertinent data regarding routine health information and patient care strategies, including professionals’ assessments and opinions on patients, evaluation records, tests, reports, subjective notes, or professionals’ contemplation.^9^ The act of documenting routine practices is crucial for ensuring the uninterrupted provision of patient care.^9^

In this practice review, we have assessed the adherence of health educators’ practice with the clinical guidelines set by the Primary Health Care Corporation (PHCC). Additionally, we have quantified the appropriateness of clinical documentation done by the educators. In this paper, we have discussed how we identified the causes of the problems, followed by using Strengths, Weaknesses, Opportunities, and Threats Analysis (SWOT analysis) to identify potential interventions selected to fill the gaps, and how we implemented action plans. The post-intervention analysis is also presented and discussed in detail in this paper.

## Methodology

**Figure 1.**
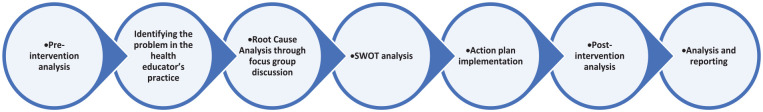
Process flow chart.

The multi-step process (Figure 1) involved conducting a preliminary assessment or pre-intervention analysis to identify any gaps in the practice, followed by a focus group discussion to identify the root causes of the problem. Subsequently, a SWOT analysis was conducted to determine potential opportunities to develop and implement interventions that can bridge the gaps and improve the quality of practice. Lastly, a post-intervention re-audit was carried out to evaluate any changes observed in the measured parameters during the entire cycle of the audit.

## Pre-Intervention Phase

During pre-intervention phase of the study, we gathered baseline data for a comprehensive assessment. The data was obtained through a thorough examination of medical records within the Clinical Information System (CIS) focused on health educators’ practice. Throughout the review process, we diligently collected the following information:

- Assessment of initial education needs.
- Social history documentation (smoking, nutritional status, exercise, drinking history etc.)
- Mental health screening using GAD 2 for Anxiety and PHQ 2 for Depression assessment.
- Problem list/diagnosis documented/reviewed.
- Health education given for the problem identified.
- Follow up advice provided.
- Follow up assessment including review of previous goals and achievement status and new complains if identified are documented.
- Follow up plan documented with new goals and follow-up instruction.

## Study Setting and Target Population

We have included the patients who have received health educators’ services from 24 health centers across Qatar. The study encompassed a sample size of 180 individuals, selected at random from data collected between April 2022 and June 2022.

### Inclusion

Patients presenting for Health Education consultation for chronic diseases at PHCC

### Exclusion criteria

Visits to Dietician, Exercise Physiologist, and Social Workers were not part of health educators’ visits and were not included in the audit population.

## Study Procedure

**Figure 2.**
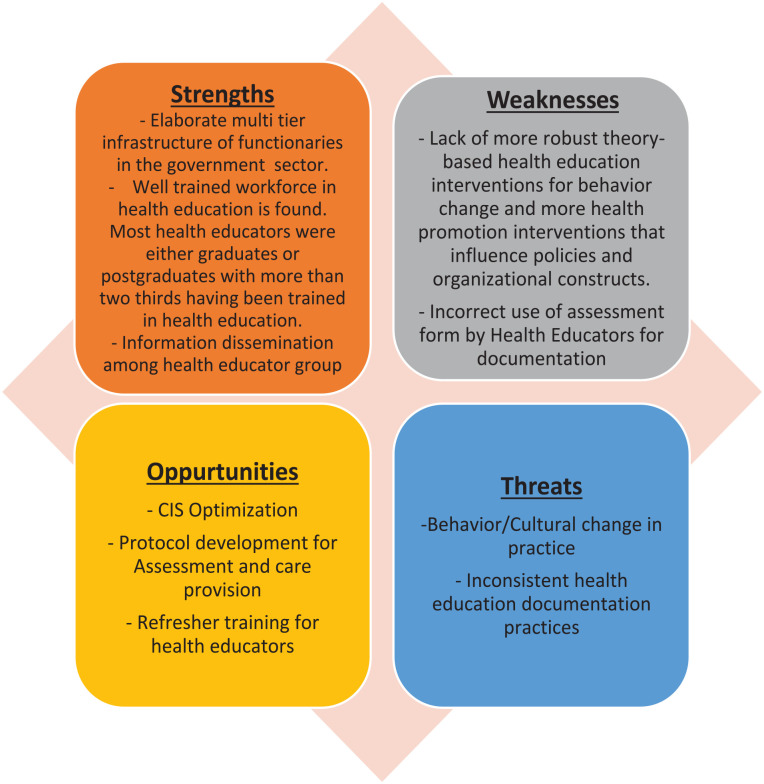
SWOT analysis.

**Figure 3.**
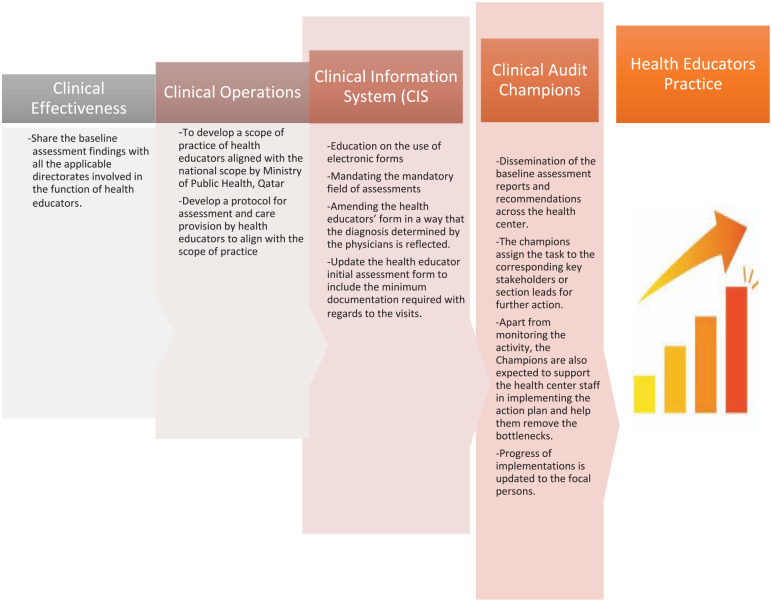
Multidisciplinary team.

1. *Situation analysis and identifying the gaps*: After conducting a thorough pre-intervention analysis (Figure 1), we have successfully identified areas of improvement within the practice. Taking into consideration the specific nature of these identified gaps, we have carefully chosen the most suitable quality improvement tools for conducting a comprehensive gap analysis.
2. *Gap analysis*: To ascertain the fundamental elements that contribute to the identified issues, we organized a focus group discussion that included the key stakeholders.
3. *Root Cause Analysis (RCA) through focus group discussion*: We engaged the primary stakeholders from the health educators’ department to discuss the audit findings, exploring and pinpointing the underlying reasons for the gaps identified. A focus group meeting was held. The results of the audit were shared and deliberated upon during the focus group meeting, where the key stakeholders were invited to identify potential causes for the problem and clinical audit team acted as a moderator. Every identified cause was documented on a presentation board. After the brainstorming session, a decision was made based on the identified causes. The causes were priorotized with the highest number of votes obtained and were chosen for SWOT analysis (Figure 2).
4. *Why SWOT analysis?*
A SWOT analysis (Figure 2) serves as the foundation for strategic planning and decision-making processes. The focus group session involving key stakeholders revealed that while there were shortcomings in the practice of health educators, there were several favorable elements in their processes that could benefit from some targeted interventions. The initial assessment results indicated an equal number of weaknesses, strengths, and opportunities. Consequently, a SWOT analysis was deemed suitable for determining the most crucial areas for intervention.
By conducting this analysis, we were able to determine the most suitable path for long-term sustainability and establish realistic objectives while efficiently managing our resources (Figure 2). The organization’s various departments and stakeholders came together to form a multidisciplinary team, contributing their insights to the SWOT analysis. Embracing a collaborative approach has fostered open communication and a shared sense of accountability among team members, enabling us to identify and implement initiatives effectively. By recognizing our strengths and weaknesses and being mindful of potential opportunities and threats, we were able to develop strategies that ensured optimal outcomes.
5. *Intervention*
The implementation of multifaceted interventions to enhance health educators’ practices had necessitated the adoption of a multidisciplinary team approach (Figure 3). These interventions were formulated after identifying the strengths devising suitable action plans against to the Weakness, Threats, and the Opportunities. The successful execution of these interventions relied on the following crucial components.

### Multidisciplinary Team

1. *Clinical operations*:

The professionals engaged in this activity include the Health Educators’ section lead and the members of the health educators’ team. The action plan that was created and put into effect consists of the following:

❖ Developed a scope of practice for health educators aligned with the national scope by Ministry of Public Health, Qatar
❖ Developed a protocol for assessment and care provision by health educators and aligned with the scope of practice.

*2. Clinical Information System (CIS)*

The professionals involved in this endeavor were subject matter experts and CIS specialists. The CIS team has executed the following action plans:

❖ Created awareness on the use of electronic forms.
❖ Incorporated mandatory field of assessment in CIS.
❖ Amended the health educators’ form in a way that the diagnosis determined by the physicians automatically reflected.
❖ Updated the health educator initial assessment form to include the minimum documentation required with regards to the visits.

*3. Clinical effectiveness section*

The professionals involved in this activity were members of the clinical audit team, clinical effectiveness directorate, and senior management representatives. The following actions were implemented:

❖ The baseline assessment findings were disseminated to all the pertinent directorates engaged in optimizing the role of health educators.
❖ The taskforce meeting was led with the participation of senior management representatives, who were engaged in executing the action plan at all the health centers.

4. *Clinical audit champions*

The professionals involved in this activity are physicians, nurses, health center section leads, the clinical audit team, and the clinical effectiveness staff.

- The clinical audit champions were comprised of physicians and nurses selected from every healthcare center to spearhead transformation within the healthcare facility.
- A focal person from the clinical effectiveness department presented audit findings with the champions. Further action plans that should be executed in the health centers were discussed. The champions were provided with guidance during this meeting.
- The champions disseminated the baseline reports and recommendations across the health center, assigning responsibilities to important stakeholders or department heads for subsequent implementation. For example, in the case that the action plan is aimed at health educators, the champions have the duty of convening a meeting with the health educator lead in order to deliberate on strategies for improving practice.
- The section head then communicated this recommendation to team members to address any obstacles and created an action plan for implementing changes.
- Any identified constraints are reported back to the champions, who resolved issues within their capacity locally and escalated those beyond to the focal person.
- In addition to overseeing activities, the champions assisted health center staff in executing the action plan and eliminating obstacles, parallelly updating focal persons on progress.
- Following updates from the champions, focal persons invited them to participate in biweekly follow-up meetings to review implementation status and address challenges, with support from the Clinical Effectiveness department as needed.

6. *Post-intervention phase*:
The follow-up phase occurred 12 months post-baseline assessment, after implementation of the intervention. For this study, a sample of 236 individuals was selected between April and June 2023, chosen randomly from the most recent 3 months of data. The information was gathered through medical record review, recorded on an Excel spreadsheet, and matched with the data collected during the baseline study from medical records.

### Data Collection

Data were extracted from the electronic health record system at 2 separate time points: Firstly before the interventions and secondly 12 months after the implementation of the interventions. Excel was used to collect and organize the data. Pivot tables were used to determine frequency of cases across variables. Excel was further used to produce histograms and other figures.

## Outcome Measures

The primary outcome of this study was as follows:

1. *Appropriate assessment and documentation of education needs*: This assessment evaluated if the health educators are properly evaluating the educational requirements of the patients.
2. *Appropriate documentation of social history*: The assessment was carried out to ascertain whether the health educators were assessing and documenting the social background of patients, including smoking status, nutrition, exercise, and so on.
3. *Mental health screening was done during the initial visit using GAD 2 for anxiety and PHQ 2 for depression assessment*: This metric evaluated the percentage of individuals who had received mental health evaluations.
4. *Problem list/diagnosis appropriately documented*: This measure assessed the proportion of patients’ files that had a problem list/diagnosis appropriately documented.
5. *Appropriate health education delivered according to the needs of the patient*: This metric evaluated the percentage of patients who were provided with suitable education by the health educators, taking into account their educational requirements assessment.
6. *Follow-up advice given*: This measure assessed the proportion of patients who received follow-up advice and appointments subsequent to the initial visit.
7. *Follow-up assessment done (including review of previous goals and achievement status, and new complaints, if identified, if were documented)*: This evaluation determined if the health educators evaluated the progress of previous objectives and their attainment. Additionally, it examined whether new concerns were identified and recorded accurately.
8. *Follow-up plan documented with new goals and follow-up instructions*: This metric evaluated the percentage of individuals who were provided with a follow-up strategy containing new goals and instructions. Additionally, a portion of evaluations identified the necessity for a consultation with a medical professional or a specialist, or the requirement for discharge.
9. *Data analysis*: The data analysis was conducted using Microsoft excel to assess the significance of changes in the assessment of education needs, social history, mental health screening, problem list/diagnosis documentation, health education, follow up advise, follow up assessment and plan for new goals.

## Statistical Analysis

The Statistical analysis was performed STATA 15.1 (College Station, TX, USA). Wilcoxon Sign rank test has been used to find Mean and Median difference in pre and post assessment of 7 criteria. *P* < .05 considered as statistically significance.

## Ethical Consideration

This study is exempted from formal ethical review as it constitutes a quality improvement project.

## Results

**Figure 4.**
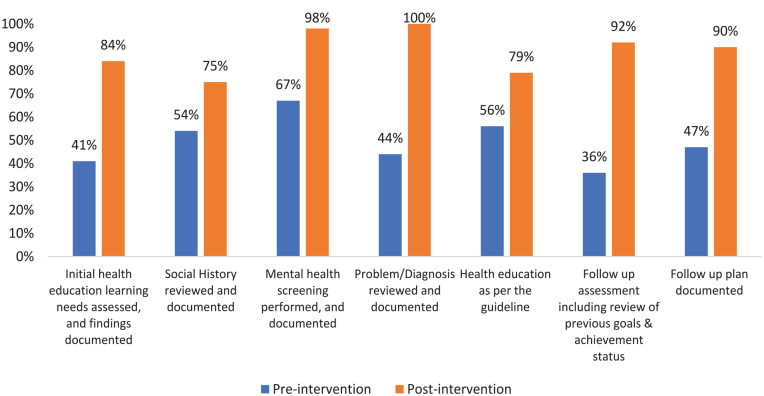
Pre and post intervention results.

Following the initial health education and learning needs assessment, the adherence rate was found to be 41%. However, after the post-intervention, there was a significant increase in compliance, reaching 81% (*P* = .001). Similarly, the social history review also showed improvement, increasing from 54% at the baseline to 75% at the post-intervention (*P* = .198). Initially, 67% of patients underwent mental health screening for anxiety using GAD2 and for depression using PHQ 2. However, following the post-intervention, the adherence rate significantly increased to 98% (*P* ≤ .001). Additionally, problem list documentation was completed for all patients, reaching an adherence rate of 100% (*P* ≤ .001), whereas it was 44% during the baseline assessment (Table 1 and Figure 4).

**Table 1.** Pre and Post Intervention analysis.

Outcome measures	Before intervention		After intervention		Absolute mean difference	*P* value
	Mean ± SD	Median (IQR)	Mean ± SD	Median (IQR)		
Initial Health Education Learning needs assessed, and findings documented.	40.56 ± 34.04	25 (60)	84.47 ± 17.56	90 (20)	43.88	.001^a^
Social History documented/reviewed	54.44 ± 33.47	60 (40)	71.11 ± 32.16	85 (50)	16.67	.198^a^
Mental Health screening done	67.22 ± 31.92	80 (40)	97.78 ± 5.48	100 (0)	30.56	<.001^a^
Problem list/diagnosis documented/reviewed.	43.89 ± 42.85	30 (90)	99.44 ± 2.36	100 (0)	55.56	<.001^a^
Health education referral order addressed appropriately providing Education specific to patient needs and condition as per the recommendation of clinical guidelines documented including follow-up Instructions if required	56.11 ± 31.46	50 (60)	77.22 ± 28.45	90 (30)	21.11	.013^a^
Health Educators Follow up assessment including Review of Previous Goals and achievement status and any new complains if identified are documented.	38.33 ± 44.21	16.5 (100)	92.67 ± 23.73	100 (0)	54.33	.001^a^
Health Educators Follow up Plan documented with (i) New goals and/or (ii) Follow-up instruction if required for follow up back to health educator or referral to Physician or any other specialty or discharge	47.24 ± 36.56		85.71 ± 29.53		38.47	.004^a^

a Wilcoxson sign rank test used to find the significance difference between the baseline and follow-up data.

In the initial evaluation, it was found that 56% of patients were provided with health education and follow-up guidance. After the intervention, however, the percentage increased to 79% (*P* = .013) of patients who received health education and follow-up advice. During the first visit, health educators establish goals for the patients, and it is crucial to evaluate the progress of these goals during the follow-up. At the baseline assessment, 35% of patients underwent follow-up assessment, which included a review of previous goals and their achievement status. In contrast, after the intervention, this figure rose significantly to 92% (*P* = .001). Similarly, 47% of patients were given a follow-up plan with new goals and instructions, but following the intervention, this number improved to 90% (*P* = .004). The overall adherence rate during the baseline assessment was 51%, but after the intervention, the practice of health educators improved to reach 88% (Table 1 and Figure 4).

Figure 4 The results obtained before and after the intervention indicate that all criteria exhibited improvement when assessed against the baseline, achieving optimal levels of compliance in the majority of the criteria.

## Discussion

Health education plays a crucial role in preventing illness and enhancing the overall well-being of individuals, serving as a key strategy in health promotion. Providing awareness about health especially to the chronic disease patients play an important role. The results of a study evaluating the impact of a multidisciplinary team approach to improving health educator practice in PHCC necessitates a discussion in the broader context of global preventive care.

Understanding that there are numerous factors that impact a health educator’s workflow, including the need of defined standard of practice, electronic medical record usage support, documentation guidelines such as the minimal amount of documentation required for each type of visits, task reconciliation, setting a benchmark for overall health educator practice at each level of the health center, and practice evaluation. These features are provided by multiple sections. The standard of practice is set by the department of health educators. The CIS division supports electronic medical record usage; the health educators department and CIS give documentation assistance; the champions create benchmarks; and the clinical effectiveness department evaluates practice. So, to strengthen the health educators practice, all of these stakeholders should collaborate. Here is where a diverse team approach is required. Collegiate cooperation, attained through effective interdisciplinary teamwork characterized by common goals, designated duties, mutual trust, streamlined communication, and quantifiable processes and outcomes, has showcased impressive accomplishments.^10^

Our baseline assessment revealed a gap in the overall health educator’s practice. Assessing the initial education and learning needs enables the educators to understand the level of knowledge patients have about their health and illnesses. To assess the health education needs of the patients, the educator has to interview the patient by asking questions related to the patient’s current health status, knowledge about the disease, especially chronic disease, awareness of related complications, and its management. Based on the assessment of patient knowledge, the health educator has to assess further health education needs and develop an education plan accordingly. The assessment details should be appropriately documented in the patient’s medical record when doing follow-up visits in the future or when consulting with different health educators in the same healthcare environment. The initial review of practice showed an adherence rate of 41% with the health education and learning needs assessment. This indicated the potential missed opportunities in assessing patients’ existing health knowledge and the chance to intervene to improve patient health outcomes.

Patient education is necessary for chronic illnesses like diabetes and hypertension to be adequately controlled and to avoid negative health effects. To obtain appropriate blood pressure management, patients with hypertension may need to learn how to take various medications as prescribed and make lifestyle changes (such as exercising or adopting a low-sodium diet).^11^ Similarly, health education also has a role in diabetes care to learn considerably more about the nuances of insulin injections, home glucose monitoring, and the diabetic diet.^12,13^ In addition, patient education is essential for helping patients accept their diagnosis and comprehend the behavioral adjustments needed to take an active part in their therapy.^14^

The results of the preliminary analysis highlighted the importance of utilizing organized approaches in addressing systemic issues. Through focused interventions guided by the SWOT analysis, we successfully pinpointed the root causes of low adherence. This method, which assessed strengths, weaknesses, opportunities, and threats, has significantly contributed to enhancing healthcare quality.^15^ A study in Belgium also employed a similar approach combining focus groups with the SWOT technique, validating the effectiveness of our strategy.^15^ The SWOT analysis indicated that the health educators team placed a significant focus on information sharing, a skilled workforce, and suitable infrastructure as their strengths. By capitalizing on opportunities such as CIS optimization, protocol advancements, and workforce training, the team effectively addressed weaknesses and threats, leading to improved performance during the post-intervention evaluation. Organizational and policy enhancements were implemented to enhance the quality of public health promotion through health education.

The baseline analysis showed inconsistency in the health educators’ initial and follow-up assessments and related documentation. Also, it shows variability in the initial learning need assessment, identifying the level of patient education, and finding barriers in order to address the issue. The root cause of this problem was uncovered as a lack of governing documents to standardize the health educator’s assessment and care. With the help of a focus group session and by involving multidisciplinary teams such as the clinical effectiveness department and clinical operations, a solution was put forward to develop a scope of practice for health educators aligned with the national scope by the Ministry of Public Health and to develop a protocol for assessment and care provision by health educators to align with the scope of practice. Further to the development of the scope of services and protocols, training was conducted for the health educators based on the new scope of services. After a year of implementing the changes, the post-intervention study shows an optimal level of adherence in assessing the initial health education learning needs; adherence increased from 41% at baseline to 84% in the post-intervention phase, likewise the assessment of social history: adherence increased from 54% to 75%, although they did not reach the optimal level of 80%.

Mental illness, such as depression, is a potential risk factor for many chronic diseases and is a poor prognostic indicator for many important chronic diseases, such as heart disease, stroke, cancer, and diabetes.^16^ Therefore, in order for an effective health educator intervention, mental health screening is essential. However, the baseline assessment showed 67% of patients undergone mental health screening with GAD 2 for Anxiety and PHQ 2 for Depression assessment tool. The low performance was considered a serious nonadherence in the health educators’ practice and the root cause of the problem was identified as a lack of awareness, and incapacity to use the assessment tool developed in CIS. As a solution to this problem training and awareness has been conducted by the health educators’ department to all the health educators across the health center, as a result in the post intervention evaluation its adherence increased significantly to reach 98%.

During the baseline evaluation, it was discovered that health educators had not appropriately documented clinical information, leading to the absence of crucial patient details required for follow-up care. The root cause of this problem was identified as the fact that clinical documentation during the initial session of health education is vast, and the educators are prone to missing important information. As a solution to this problem, a multidisciplinary team involving clinical effectiveness department, heath educators and CIS department developed a template in the CIS form which assisted the health educators to fill in all the needed information. Studies indicates that the Clinical Information System (CIS) can enhance clinical documentation and positively influence patient care outcomes.^17^

The champions are dedicated people that work with implementation of changes. Champions drew into a differing degree in various center activities, including training, support, relationship building among different sections and initiate integrated changes.^18^

In PHCC, champions across all the health centers are empowered to make decisions and make changes for improvement. To improve health educators practice, the champions-initiated awareness for all health educators on mandatory documentation by facilitating training. The clinical audit champions of each health center liaise with the health educators’ team in assessing the baseline report, identifying the gaps in their center, and initiating change plans. The champions assigned the health educators to develop their KPIs to create a sense of responsibility and ownership of their activity and insisted on the reporting of progress. The champions coordinate with a focal person in the clinical effectiveness department for further support and reporting of progress. As a result of all the change processes, the follow-up adherence to health education provided as per the guidelines improved to reach 79%, the follow-up assessment reached 92%, and the follow-up plan reached 90%. The composite compliance, which was 51% in the baseline evaluation, increased to 88% in the post-intervention phase, reflecting the overall impact of the change process.

### Limitation of This Study

The study focused exclusively on evaluating the structure and process indicators that covered the operational procedures, system, and proficiency of health educators. The analysis conducted after the intervention solely investigated if the quality improvement approach implemented improved adherence with the clinical practice guideline. Nevertheless, this study did not assess the outcome measure associated with patient outcomes.

## Conclusion

The multi-layered process encompassing pre-intervention analysis followed by implementation of interventions through involvement of a multidisciplinary team played a crucial role in improving the overall performance of the health educators. The study results showed this collaboration of different departments has positively impacted the performance of the health educators. The health educators’ practice success is dependent on collaborative working through successful multidisciplinary team-based care, which is defined by shared objectives, clear responsibilities, mutual trust, good communication, and quantifiable procedures and outcomes, with health educators at the forefront of practice.

The study has helped in bringing the importance of multidisciplinary teams into the limelight for improving health educators’ practice. Additional research is recommended to explore and evaluate the influence of a multidisciplinary team on the practice of health educators, encompassing the effectiveness of such interventions on outcome measures such as impact on prevention of chronic health diseases, as currently there is a lack of sufficient evidence in this area. By conducting further studies, we can gain a better understanding of how a diverse team approach can impact the effectiveness of clinical care.
